# Differential Adjuvant Activities of TLR7 and TLR9 Agonists Inversely Correlate with Nitric Oxide and PGE_2_ Production

**DOI:** 10.1371/journal.pone.0123165

**Published:** 2015-04-13

**Authors:** Jinhee Lee, Nuria Martinez, Kim West, Hardy Kornfeld

**Affiliations:** Department of Medicine, University of Massachusetts Medical School, Worcester, Massachusetts, United States of America; Boston University, UNITED STATES

## Abstract

Activation of different pattern recognition receptors causes distinct profiles of innate immune responses, which in turn dictate the adaptive immune response. We found that mice had higher CD4^+^ T cell expansion to an immunogen, ovalbumin, when coadministered with CpG than with CL097 *in vivo*. To account for this differential adjuvanticity, we assessed the activities of CpG and CL097 on antigen-specific CD4^+^ T cell expansion *in vitro* using an OT-II CD4^+^ T cell/bone marrow-derived dendritic cell (DC) co-culture system. Unexpectedly, ovalbumin-stimulated expansion of OT-II CD4^+^ T cells *in vitro* was potently suppressed by both TLR agonists, with CL097 being stronger than CpG. The suppression was synergistically reversed by co-inhibition of cyclooxygenases 1 and 2, and inducible nitric oxide (NO) synthase. In addition, stimulation of OT-II CD4^+^ T cell/DC cultures with CL097 induced higher levels of CD4^+^ T cell death than stimulation with CpG, and this CD4^+^ T cell turnover was reversed by NO and PGE_2_ inhibition. Consistently, the co-cultures stimulated with CL097 produced higher levels of prostaglandin E_2_ (PGE_2_) and NO than stimulation with CpG. CL097 induced higher PGE_2_ production in DC cultures and higher IFN-γ in the OT-II CD4^+^ T cell/DC cultures, accounting for the high levels of PGE_2_ and NO. This study demonstrates that the adjuvant activities of immunostimulatory molecules may be determined by differential induction of negative regulators, including NO and PGE_2_ suppressing clonal expansion and promoting cell death of CD4^+^ T cells.

## Introduction

The list of infectious agents prevented by vaccines is growing, assisted by advances in antigen and adjuvant discovery [1]. Despite this progress, there is an unmet need for effective vaccines against some of the deadliest infectious diseases including tuberculosis (TB), malaria, and AIDS. Aluminum salts (Alum) have long been the only adjuvant in vaccines approved for human use. Alum efficiently elicits antibody responses [2], but is a poor inducer of cell-mediated immunity [3], which is necessary for protection against intracellular pathogens [4]. New adjuvants have been licensed for human use, such as MF59, AS03, and AS04, and many adjuvant candidates are under development to meet the demand for diverse types of adaptive immune activation [5,6].

Vaccine adjuvants not only enhance the quantitative magnitude of adaptive immune responses, but also shape their qualitative characteristics [1]. Thus, a protective mode of adaptive immunity required against a specific pathogen could be improved by rational adjuvant formulation. Adjuvant effects are mediated by the innate immune response [7,8] and dendritic cells (DCs) are the key immune cells bridging innate and adaptive immunity [9,10]. Engagement of pattern recognition receptors (PRRs) on DCs by pathogen-associated molecular patterns (PAMPs) in adjuvant formulations initiates key signaling cascades involving transcription factors, including nuclear factor (NF)-κB, mitogen-activated protein kinase, interferon regulatory factor (IRF)-3, and IRF-7 [11,12]. This results in the induction of proinflammatory cytokines and major histocompatibility complex (MHC) molecules and costimulatory molecule expression that endow DCs with the ability to prime, expand and polarize naïve T cells [13,14]. Because individual PRR ligands have been evaluated independently without an experimental standard or any cross-comparison, the relative adjuvant activities of different PRR agonists have not been established [15]. This precludes appropriate selection of adjuvants optimized for specific vaccines.

Toll-like receptors (TLRs) are the most studied PRR in terms of adjuvant development. Particular TLR ligands activate DCs differently, which may lead to differences in the quality and quantity of adaptive immune responses [6,13,16]. Synthetic oligodeoxynucleotides (ODN) containing unmethylated CpG motifs are agonists for TLR9 [17], and are the most studied class of TLR agonists as adjuvants [15,18]. Imidazoquinolines, ligands for TLR7 and TLR8 [19–21], exert antiviral activity when topically applied on human papilloma virus-induced warts [22], and adjuvant effects for protein antigens and DNA vaccines [23–25]. TLR7/8 and TLR9 are both endosomal receptors and the signaling pathways downstream of these receptors are MyD88-dependent [26].

Defining relative adjuvant effects of different TLR ligands on T cell activation may help optimize their selection for different infectious diseases. To that end, we compared the TLR9 agonist CpG1826 and the TLR7 agonist CL097 (a highly soluble derivative of R848 [20]) for their stimulatory effects on DC and antigen-specific CD4^+^ T cell activation. We found that CpG was the more potent adjuvant in terms of enhancing antigen-specific CD4^+^ T cell expansion. The lower adjuvanticity of CL097 was attributable to higher induction of the negative regulators of T cell activation, prostaglandin (PGE_2_) and nitric oxide (NO) relative to CpG. This study provides evidence that individual TLR agonists elicit different levels of innate responses resulting in differential CD4^+^ T cell responses and that expression of negative regulators is a major determinant of the magnitude of cell-mediated immune responses. In addition, these counter-regulatory effects of TLR stimulation suggest the possible use of inhibitors of inflammatory metabolites in vaccination strategies designed to augment immunogenicity and protective efficacy.

## Materials and Methods

### Reagents

The inducible nitric oxide synthase (iNOS) inhibitor N^G^-methyl-L-arginine acetate salt (L-NMMA) and the cyclooxygenase (COX)-1/COX-2 inhibitor indomethacin (Indo) were purchased from Sigma (St. Louis, MO). An H-2b-restricted ovalbumin (OVA) class II epitope (323–339) was purchased from AnaSpec (Fremont, CA). TLR ligands, CpG1826 and CL097, were purchased from Invivogen (San Diego, CA). All the fluorescent-labeled antibodies were purchased from eBioscience (San Diego, CA). Recombinant mouse IL-2 was purchased from R & D systems (Minneapolis, MN) and neutralizing anti-IL-2 mAb (clone:JES6-1A12) was from Biolegend (San Diego, CA).

### Mouse immunization

**Fig 1:**
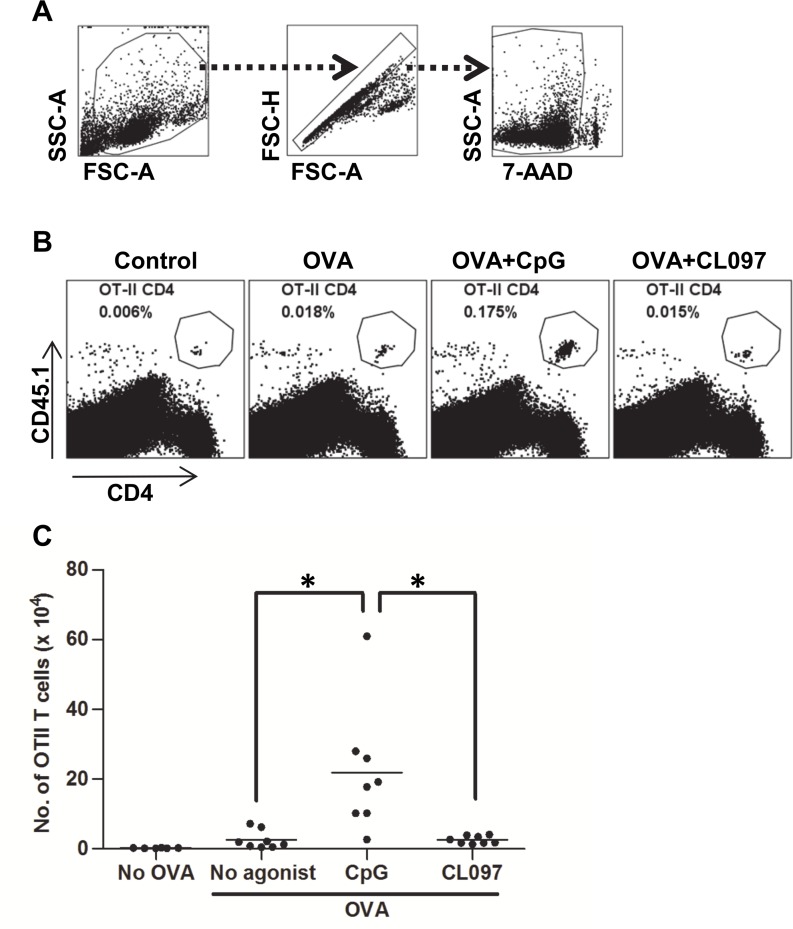
Stronger expansion of OT-II CD4^+^ T cells in mice immunized with OVA plus CpG than with OVA plus CL097. Mice were adoptively transferred with 1 x 10^6^ splenocytes obtained from OT-II mice and, 24 h later, immunized with OVA protein (25 μg) with or without CpG (20 μg) or CL097 (20 μg) formulated in IFA. Mice were sacrificed 11 days post immunization, and the number of expanded OT-II CD4^+^ T cells were quantified by cell surface staining of CD4 and CD45.1 followed by flow cytometric analysis. (A) A series of gating steps depicted in arrows involved gating on total leukocytes (FSC-A/SSC-A), on singlets (FSC-A/FSC-H), and on live cells (7-AAD/SSC-A). (B) Each FACS plot is an analysis of live splenocytes for one of eight mice within a group. Cells double positive for CD4 and CD45.1 demarcated in the plot are OT-II CD4^+^ T cells. Numbers are the percentages of the OT-II CD4^+^ T cells in spleens. (C) The total numbers of OT-II CD4^+^ T cells in spleens were calculated based on the percentages of OT-II CD4^+^ T cells and known numbers of splenocytes. Statistical differences were analyzed by one-way ANOVA between treatment groups. Data shown are representative of three independent experiments. *p<0.002.

Mice were housed in pathogen-free environment at Animal Medicine facility at the University of Massachusetts Medical School (UMMS). All animal experiments were carried out in strict accordance to the guidelines set forth by the National Institutes of Health regarding the use of laboratory animals under protocols approved by the Institutional Animal Care and Use Committee (A-1928) at UMMS. Mice were given irradiated food and acidified autoclaved water *ad libitum*, and the health and well-being of animals were closely monitored by researchers and veterinary technicians. Mice were euthanized by CO_2_ narcosis followed by cervical dislocation. Wild-type C57BL/6 mice expressing the CD45.2 congenic marker (The Jackson Laboratory, Bar Harbor, ME) were adoptively transferred intravenously with one million splenocytes obtained from the congenic OT-II transgenic mice. More than 99% of splenocytes isolated from male OT-II mice express CD45.1 and are transgenic to H-2^b^-restricted OVA class II epitope (323–339). 24 h later, mice were immunized subcutaneously with Incomplete Freund’s adjuvant (IFA) (Sigma) containing 25 μg of OVA protein with or without 20 μg of CpG or CL097. Equal volumes of IFA and Ag/CpG or Ag/CL097 solution were mixed and 100 μl of the formula was administered to each mouse. Animals were sacrificed after 11 days, and splenocytes were counted and stained with Alexa 450-labeled anti-CD45.1 and *allophycocyanin* (APC) anti-CD4 antibodies. Cells were gated on singlets based on FSC-A/FSC-H, and live cells based on FSC/SSC and 7-amino-actinomycin D (7-AAD) (Fig 1A). Flow cytometry was performed on an LSRII flow cytometer (BD Biosciences, San Jose, CA), 500,000 leukocyte-gated events were collected and data analyzed with FlowJo PC software (TreeStar, Inc.). The number of OT-II CD4^+^ T cells from a spleen was calculated based on the number of total splenocytes and the percentage of CD45.1^+^/CD4^+^ cells.

### Generation of BMDCs

Bone marrow cells were harvested from C57BL/6 mice. BMDCs were prepared by culturing bone marrow cells in 10 ml of RPMI1640 medium (Gibco, Gaithersburg, MD) supplemented with 10 ng/ml granulocyte-macrophage colony-stimulating factor (GM-CSF, R&D Systems), 10% fetal bovine serum (FBS) (BioWhittaker, Walkersville, MD), 100 units/ml of penicillin, 100 mg/ml of streptomycin, and 2 mM glutamine, and 50 μM 2-mercaptoethanol (complete media) for 8 days. Fresh media was added on days 2 and 5 to maximize DC yield. Non-adherent DCs were collected from 100 mm petri dishes by pipetting, counted and plated.

### IL-2 and IFN-γ measurement

5 x 10^4^ OT-II CD4^+^ T cells were co-cultured with 1 x 10^4^ DCs in flat-bottom 96-well plates. The co-cultures were stimulated with OVA protein (25 μg/ml) or OVA peptide (0.1 μM), plus CpG (1 μg/ml) or CL097 (1 μg/ml) for four days. Supernatants from cultures were collected and stored in -70°C until use. IL-2 and IFN-γ were measured by a Duoset ELISA kit from R & D (Minneapolis, MN).

### PGE_2_ measurement

To measure PGE_2_ produced in DCs stimulated with TLR agonists, DCs were plated in 48-well plates at 2 x 10^5^ cells per well in 1 ml of complete media in the presence or absence of CpG or CL097 for two days. To measure PGE_2_ produced in OT-II CD4^+^ T cell/DC cultures, purified 5 x 10^4^ OT-II CD4^+^ T cells and 1 x 10^4^ DCs were plated in flat-bottom 96-well plates in 200 μl of complete media in the presence or absence of OVA protein (25 μg/ml) with CpG or CL097 for 3 days. Supernatants from cultures were collected and stored in -70°C until use. PGE_2_ was measured by a competitive ELISA kit from Enzo (Farmingdale, NY).

### Nitric oxide measurement

To determine NO levels, nitrite (NO_2_^-^) was measured using the Nitric Oxide Quantitation Kit purchased from Active Motif (Carlsbad, CA). 4 x 10^4^ OT-II CD4^+^ T cells and 1 x 10^4^ BMDCs were plated in 96-well flat-bottom plates and stimulated with OVA protein (25 μg/ml) in the presence or absence of CpG or CL097 (1 μg/ml) at a total volume of 200 μl for four days. Supernatants were harvested and diluted by three fold with PBS containing 0.05% Tween-80 before the assay. Nitrite was measured according to the manufacturer’s protocol. Briefly, 100 μl of diluted supernatants were mixed with 50 μl of Griess reagent A and 50 μl of Griess reagent B, and the color was allowed to develop at room temperature for 10 min. Nitrite standard was serially diluted ranging from 0.07 to 10 μM. The optical density of the color was measured at 540 mm with a spectrophotometer.

### OT-II T cell proliferation assay

Splenocytes harvested from OT-II transgenic mice were stained with 2 μM CFSE, and then CD4^+^ T cells were purified by magnetic separation (Miltenyi Biotec, Auburn, CA). 5 x 10^5^ OT-II CD4^+^ T cells were co-cultured with 1 x 10^5^ DCs in 48-well plates. The CD4^+^ T cell/DC co-cultures were stimulated with OVA protein (25 μg/ml) or OVA peptide (0.1 μM), plus CpG (1 μg/ml) or CL097 (1 μg/ml) in the presence or absence of L-NMMA (40 μM) or Indo (10 μM) for 3–5 days as described in figure legends. For 14-day proliferation assays, 5 x 10^3^ OT-II CD4^+^ T cells were incubated with 1 x 10^5^ BMDCs and stimulated with OVA antigen and TLR agonists in the presence or absence of the inhibitors for 14 days. At the end of the culture period, 4 x 10^5^ splenocytes (~60,000 CD4^+^ T cells) obtained from wild-type C57BL/6 mice (CD45.2) were added to the cultures immediately before harvest. Cells were stained with Alexa 450 anti-CD45.1 and APC anti-CD4 to calculate the ratio of OT-II CD4^+^ T cells and wild-type CD4^+^ T cells. The formula to calculate OT-II CD4^+^ T cells is: (%of OT-II CD4^+^ T cells ÷ % of CD45.1^-^ CD4 T cells) x 60,000. Dead cells were excluded by staining with 7-AAD.

### Intracellular staining

To enhance the sensitivity of intracellular cytokine detection, BD GolgiPlug (BD Biosciences) was added 5 h before the end of culture according to the manufacturer’s recommendation. Cells were stained with PerCP-Cy5.5 anti-CD4 and then with LIVE/DEAD Aqua Dead cell stain (Invitrogen, Grand Island, NY) to gate on live CD4^+^ T cells. Cells were then fixed and permeabilized using BD Cytofix/Cytoperm (BD Biosciences) according to the manufacturer’s protocol. After permeabilized, cells were stained with APC anti-IFN-γ antibody and analyzed by flow cytometry.

For intracellular staining for FoxP3, cells were first stained with PE anti-CD4, APC anti-CD25 antibodies, and Aqua Dead cell stain, and fixed and permeabilized with FoxP3 staining buffer set according to the manufacturer’s instructions (eBioscience). After permeabilization, cells were stained with Alexa450 anti-FoxP3 antibody.

### Measurement of CD4^+^ T cell death

To measure the cell viability of purified OT-II CD4^+^ T cells, 5 x 10^5^ OT-II CD4^+^ T cells stained with or without CFSE were co-cultured with 1 x 10^5^ DC for 3–4 days. To measure the cell viability of wild-type CD4^+^ T cells, splenocytes obtained from wild-type CD57BL/6 mice were co-cultured with 1 x 10^5^ DC for 3 days. At the end of experiment, cells were stained with APC anti-CD4 antibody and 7-AAD, and analyzed by flow cytometry.

### *In vivo* treatment with indomethacin and aminoguanidine hemisulfate

Indomethacin stock solution in DMSO was diluted in PBS for injection. Mice were injected i.p. with Indo at a dose of 2.5 mg/Kg in 200 μl three times 4 days apart starting from the day of immunization [27]. Control mice were injected with PBS containing the same amount of DMSO. Aminoguanidine hemisulfate (AG) was dissolved in drinking water at 2.5% along with 1% glucose. Mice were given drinking water containing AG for 8 days starting from the day of immunization [28]. Control mice were given drinking water containing 1% glucose.

### Statistical analysis

Statistical analysis was performed using Graph Pad Prism v.5.02 (Graphpad Software Inc., La Jolla, CA) software. Unless otherwise stated, data from independent experiments are shown as mean ± SD. Comparisons between groups were evaluated with Student’s t-test or One-way analysis of variance (ANOVA) test followed by Dunnett's Multiple Comparison Test. A *p*-value ≤ 0.05 was regarded as statistically significant.

## Results

### CpG promotes greater antigen-specific CD4^+^ T cell expansion *in vivo* than CL097

To investigate if stimulation of different TLRs in vaccination has an impact on the magnitude of the cellular immune response, we compared the adjuvant candidates CpG and CL097 for their ability to enhance CD4^+^ T cell responses. Increasing CD4^+^ T cell expansion has been proposed as one approach to enhance memory cell generation and vaccine efficacy [29]. For sensitive measurement of CD4^+^ T cell expansion *in vivo*, we adoptively transferred splenocytes containing OVA-specific T cell receptor transgenic CD4^+^ T cells obtained from OT-II mice into wild-type C57BL/6 recipients and monitored their expansion after immunization. OT-II CD4^+^ T cells carry the congenic marker CD45.1 that is used to distinguish OT-II CD4^+^ T cells from CD4^+^ T cells of the recipient wild-type mice, which express CD45.2. One day after OT-II CD4^+^ T cell transfer, recipient mice were immunized subcutaneously with OVA protein in the presence or absence of CpG or CL097 formulated in IFA. The water-in-oil emulsion IFA was chosen as a delivery vehicle for the unbiased packaging of CpG and CL097. 11 days after immunization, harvested splenocytes were stained for CD4 and CD45.1 followed by flow cytometric analysis to enumerate the OT-II CD4^+^ T cells. OT-II CD4^+^ T cells significantly expanded by immunization with OVA alone compared to the control of IFA without antigen, but the addition of CpG to OVA further increased CD4^+^ T cell expansion over OVA alone (Fig 1B and 1C). In contrast, adding CL097 to the formula containing OVA did not significantly increase CD4^+^ T cell expansion over OVA alone (Fig 1B and 1C). This poor adjuvant activity of CL097 was not due to the weak *in vivo* activities of CL097 in the preparation because the CL097 formula enhanced OT-II CD4^+^ T cells comparable to the CpG formula up until four days post immunization (S1 Fig). This observation suggests that there are differences among TLR agonists in their activity as adjuvants to stimulate CD4^+^ T cell expansion.

### OT-II CD4^+^ T cell proliferation is suppressed by TLR agonists independent of IL-2

**Fig 2:**
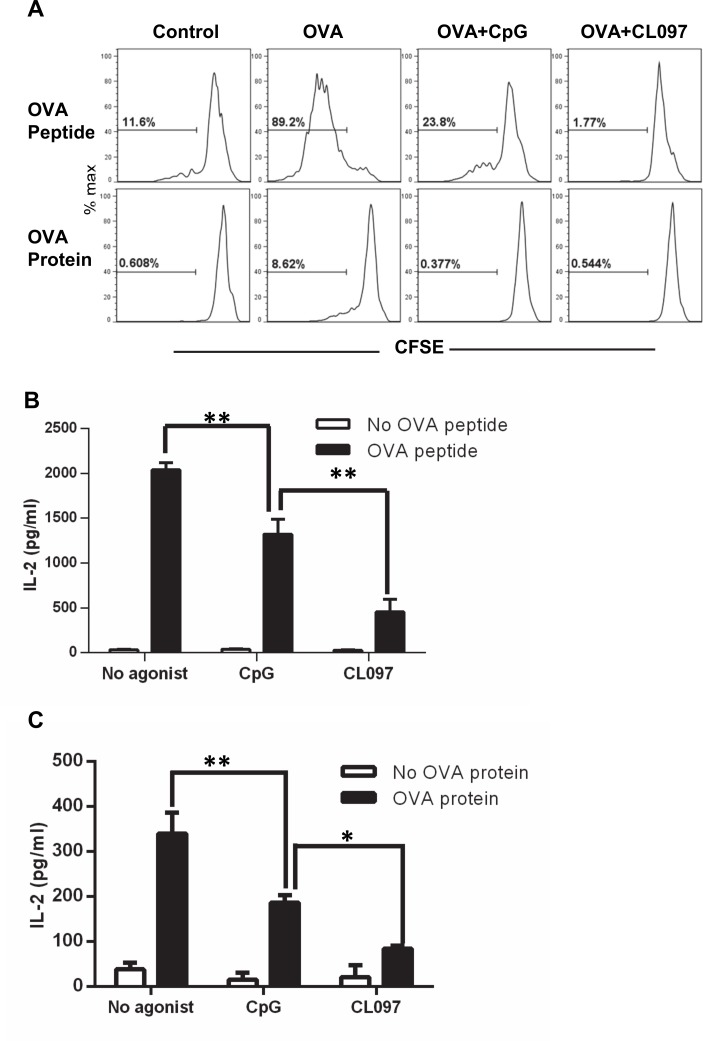
Stimulation of DCs with CpG and CL097 suppresses OT-II CD4^+^ T cell proliferation and IL-2 production. OT-II CD4^+^ T cell/DC co-cultures stimulated with TLR ligands and OVA peptides were harvested to assess CD4^+^ T cell proliferation (A) and IL-2 production (B and C). (A) OT-II CD4^+^ T cells stained with CFSE were co-cultured with DCs and stimulated with OVA peptide (0.1 μM) or OVA protein (25 μg/ml) in the presence or absence of CpG or CL097 (1 μg/ml) for four days until analyzed by flow cytometry. The range and number in each histogram represent cells that proliferated at least one time. Supernatants from cultures stimulated with OVA peptide (B) or OVA protein (C) in the presence or absence of CpG (1 μg/ml) or CL097 (1 μg/ml) for four days were harvested to measure IL-2 levels by ELISA. Statistical differences between treatments in OVA stimulated cells were analyzed by one-way ANOVA. Data are expressed as mean ± SD of triplicate wells. Data shown are representative of three independent experiments. *p<0.05; **p<0.005.

**Fig 3:**
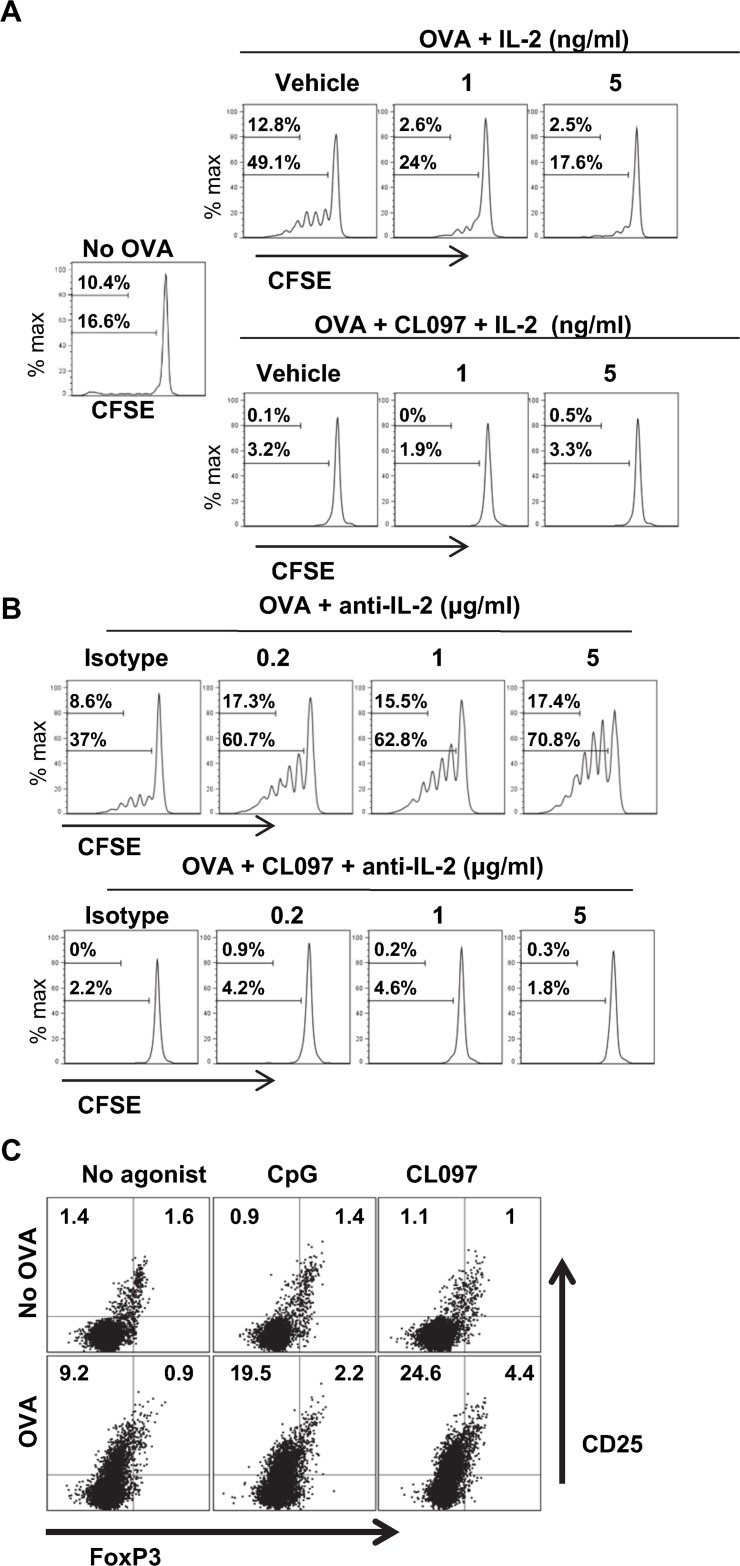
Suppression of OT-II CD4^+^ T cell expansion by TLR agonists is IL-2 independent. OT-II CD4^+^ T cells/DC co-cultures were stimulated with OVA protein (25 μg/ml) in the presence or absence CL097 (1 μg/ml) for 5 days. (A) Various concentrations of recombinant IL-2 or (B) neutralizing anti-IL-2 antibody were added to the cultures during stimulation. The two sets of ranges and numbers in each histogram represent cells that proliferated more than three times (upper) and at least one time (lower). (C) The cultures were stimulated with OVA protein in the presence or absence of CpG or CL097 for three days, and the cells were stained for CD25 and FoxP3 to measure expression levels of CD25 on FoxP3^-^ and FoxP3^+^ CD4^+^ T cells. Data shown are representative of three independent experiments.

To further investigate the direct effects of the TLR agonists on the DC function to prime CD4^+^ T cells, we developed an *in vitro* priming assay using OT-II T cells. Congenic DCs from wild-type C57BL/6 mice and OT-II CD4^+^ T cells were stimulated with CpG or CL097 in the presence of OVA protein or peptide and then CD4^+^ T cell expansion was assessed by CFSE dilution. It has been shown that DCs differentiated from bone marrow progenitor cells with GM-CSF are efficient antigen-presenting cells because they constitutively express high levels of costimulatory molecules [9]. Consistent with that report, we found that DCs loaded with OVA peptide and, to a lesser extent, OVA protein induced CD4^+^ T cell proliferation in the absence of TLR stimulation (Fig 2A). Addition of CpG or CL097 suppressed CD4^+^ T cell proliferation induced by both OVA peptide and protein alone (Fig 2A), which was unexpected given their *in vivo* effects. This suppression in the *in vitro* priming assay was more strongly mediated by CL097 than CpG when OVA peptide was used (Fig 2A) and directly correlated with reduced production of the T cell growth factor IL-2 (Fig 2B). Although T cell expansion in response to OVA protein was completely blocked both by CpG and CL097 (Fig 2A), IL-2 production was lower in cultures stimulated with CL097 (Fig 2C). Reconstitution with exogenous IL-2 failed to restore CD4^+^ T cell proliferation in DC co-cultures with OVA and CL097 present, indicating that the suppressive effect of CL097 did not result from relative IL-2 deficiency (Fig 3A). Addition of exogenous IL-2 decreased CD4^+^ T cell expansion in cultures stimulated with OVA only, suggesting a regulatory role for IL-2 in this culture system. Consistent with that model, neutralizing anti-IL-2 mAb potently enhanced the CD4^+^ T cell proliferation induced by OVA alone (Fig 3B). Blocking IL-2 failed to enhance CD4^+^ T cell expansion suppressed by CL097, suggesting that IL-2 is not involved in the suppression by CL097. The absence of IL-2 responsiveness is not due to reduction of the IL-2 receptor CD25 because the addition of CpG and CL097 further increased the CD25 levels induced by OVA stimulation (Fig 3C). Stimulation with CpG and CL097 increased not only effector cells but also FoxP3^+^ regulatory T cells.

### Suppression of OT-II T cell proliferation by TLR agonists is mediated by prostaglandins and NO

**Fig 4:**
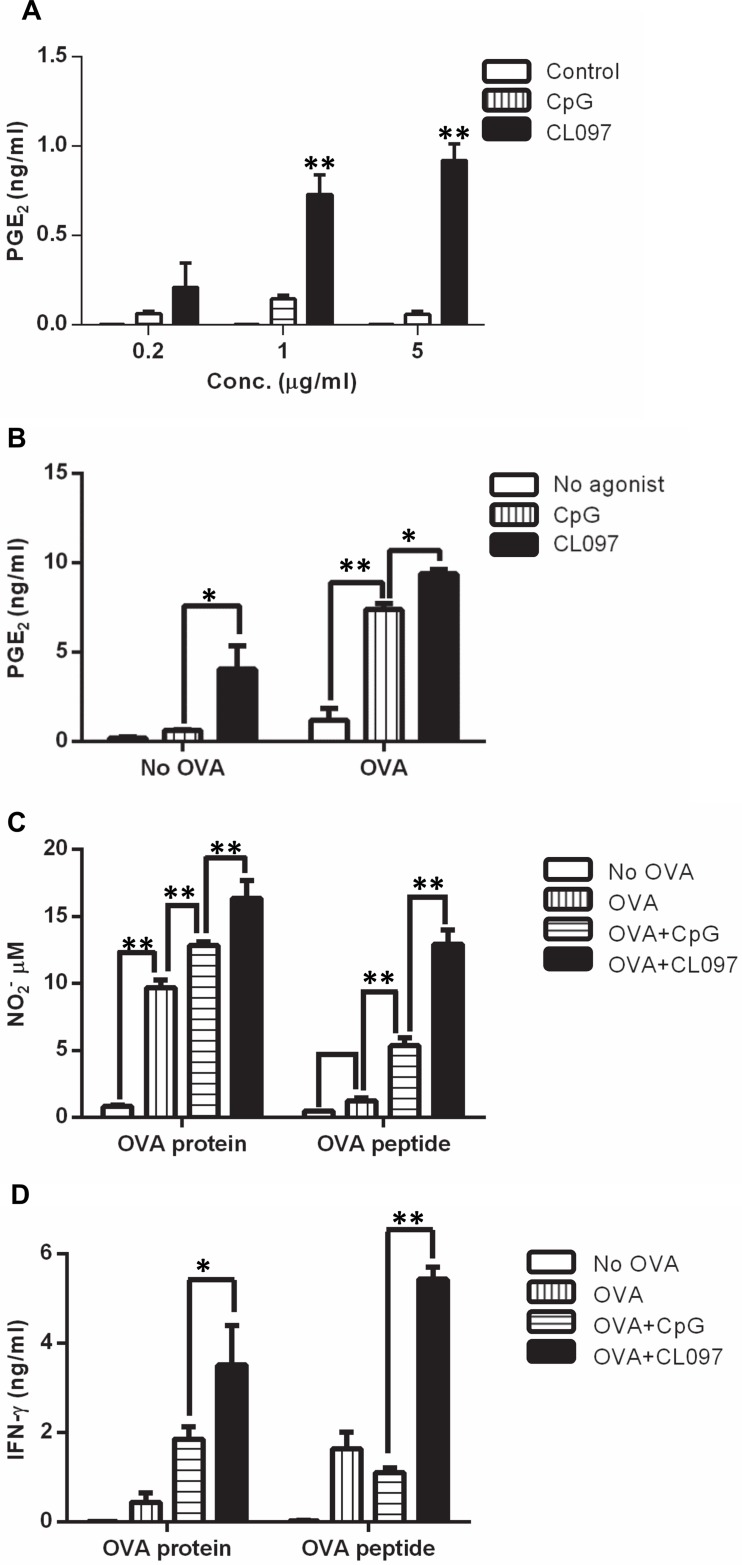
CL097 induces higher levels of PGE_2_, NO_2_^-^ and IFN-γ than CpG. (A) DCs were stimulated with CpG or CL097 at concentrations of 0.2, 1, and 5 μg/ml for 2 days, and PGE_2_ levels were measured from culture supernatants. Data shown are representative of two independent experiments. (B) OT-II CD4^+^ T cell/DC co-cultures were stimulated with OVA protein (25 μg/ml) in the presence or absence of 1 μg/ml CpG or CL097 for 3 days, and PGE_2_ levels were measured. Data shown are representative of two independent experiments. (C) OT-II CD4^+^ T cells/DC co-cultures were stimulated in the presence or absence of TLR agonists and OVA protein (25 μg/ml) for four days, and supernatants were collected to measure NO_2_^-^ by Griess assay. Data shown are representative of three independent experiments. (D) OT-II CD4^+^ T cells/DC co-cultures were stimulated in the same conditions mentioned above for 4 days, and supernatants were collected to measure IFN-γ by ELISA. Data shown are representative of three independent experiments. Statistical differences between treatments were analyzed with one-way ANOVA. The graph shows the mean ± SD of triplicate wells. *p<0.05; **p<0.005.

**Fig 5:**
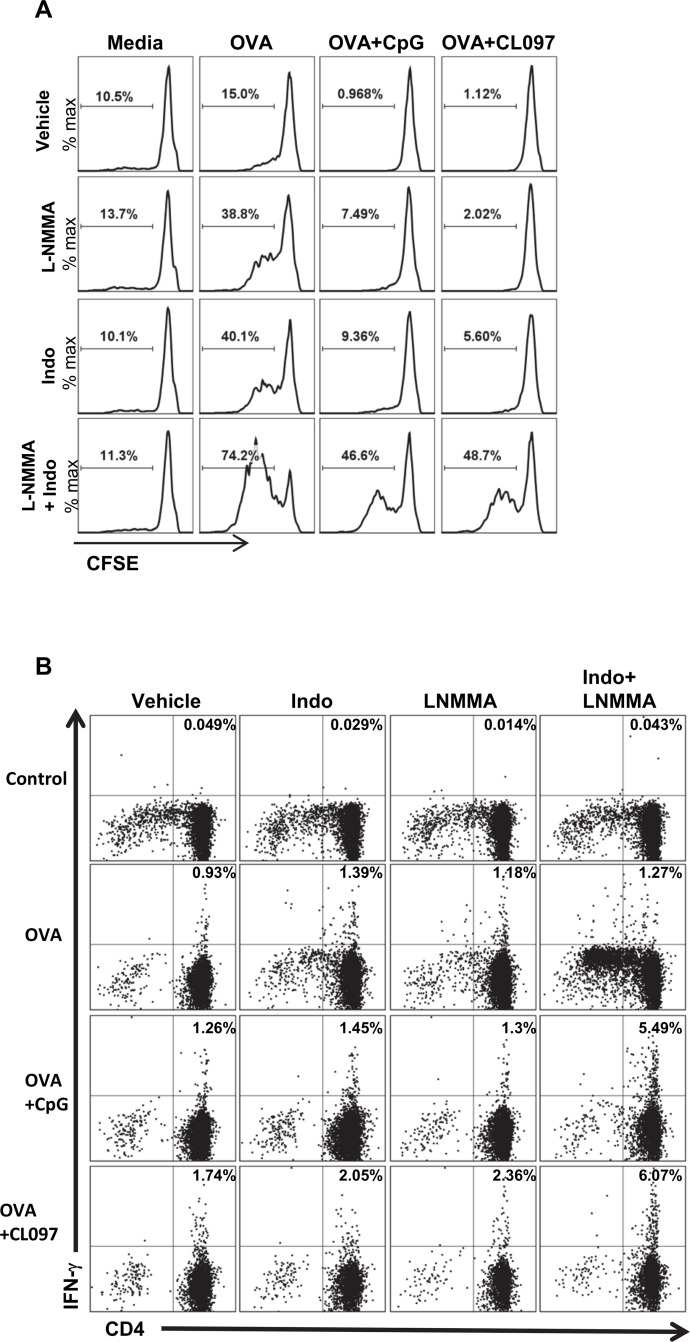
Inhibition of COX-1/COX-2 and iNOS synergistically enhances OT-II CD4^+^ T cell proliferation and IFN-γ production *in vitro*. (A) CFSE-stained OT-II CD4^+^ T cells were mixed with congenic DCs and stimulated with OVA protein with or without TLR agonists in combination with L-NMMA (40 μM) or Indo (10 μM) for 4 days. Proliferation of OT-II CD4^+^ T cells were analyzed by CFSE dilution. The range and number in each plot indicate cells that proliferated at least one time. Data shown are representative of two independent experiments. (B) OT-II CD4^+^ T cells were mixed with congenic DCs and stimulated with OVA protein with or without TLR agonists and inhibitors for 4 days. GolgiPlug was added to the cultures to inhibit exocytosis of intracellular IFN-γ at 5 h before harvest. Cells were first stained for CD4, fixed, and stained for intracellular IFN-γ. The numbers in the upper right quadrant signifies the percentage of IFN-γ positive cells among CD4^+^ T cells. Dead cells were excluded using a fixable live/dead dye. Data shown are representative of two independent experiments. iNOS: inducible nitric oxide synthase; COX: cyclooxygenase.

Since PGE_2_ and NO are known to suppress T cell proliferation *in vitro* [30–39], we speculated that they might play a role in the suppression of CD4^+^ T cell expansion induced by CpG and CL097. We measured secreted PGE_2_ levels from DC cultures and from OT-II CD4^+^ T cell/DC co-cultures. PGE_2_ levels were significantly higher in DCs stimulated with CL097 than with CpG (Fig 4A). PGE_2_ levels were also found to be higher in OT-II CD4^+^ T cell/DC co-cultures stimulated with CL097 in the presence of OVA protein (Fig 4B). Consistent with the result from DC cultures, PGE_2_ levels were higher in the co-cultures stimulated with CL097 in the absence of OVA protein (Fig 4B). This indicates that the tendency of CL097 to induce high PGE_2_ production occurs at both innate and adaptive levels. To determine NO levels, co-cultures of OT-II CD4^+^ T cells and DCs were stimulated with OVA protein or OVA peptide in the presence or absence of CpG or CL097 for four days, and culture supernatants were harvested to measure NO_2_^-^ accumulation. CL097-stimulated cultures had a higher concentration of NO_2_^-^ than CpG-stimulated and OVA alone cultures (Fig 4B). Consistent with the fact that IFN-γ is the major inducer of NO [40] and PGE_2_ [41], IFN-γ levels were highest in cultures stimulated with CL097 (Fig 4C). This implied that higher levels of IFN-γ/NO/ PGE_2_ may have been responsible for higher suppression of CD4^+^ T cell proliferation by CL097 *in vitro* and possibly for higher CD4^+^ T cell expansion in mice immunized with CpG stimulation than CL097 stimulation. To confirm that PGE_2_ and NO were responsible for the suppression of CD4^+^ T cell expansion *in vitro*, we tested the effects of the COX-1/COX-2 inhibitor Indo and the iNOS inhibitor L-NMMA. Co-cultures of DCs and OT-II CD4^+^ T cells were pulsed with OVA protein alone or OVA protein plus CpG or CL097 in the presence or absence of Indo or L-NMMA. The iNOS inhibitor minimally enhanced CD4^+^ T cell expansion in CpG and CL097-treated cultures, and treatment with Indo alone produced a modest increase in T cell expansion induced by OVA-pulsed DCs stimulated with CpG or CL097 (Fig 5A). Of note, the combination of L-NMMA and Indo synergistically enhanced antigen-stimulated T cell proliferation in the presence of TLR ligands (Fig 5A), indicating that CpG and CL097 suppress T cell expansion through production of both NO and PGE_2_. Treatment either with L-NMMA or Indo substantially enhanced CD4^+^ T cell expansion in cultures stimulated with OVA alone, indicating that the moderate amounts of NO and PGE_2_ induced by OVA (Fig 4) are functionally significant. In addition to the proliferative response, Indo and L-NMMA synergistically enhanced IFN-γ production induced by OVA plus CpG or OVA plus CL097 as demonstrated by intracellular cytokine staining (Fig 5B). On the other hand, IFN-γ production induced by OVA alone was not enhanced by either inhibitor. These results suggest that PGE_2_ and NO stimulated by TLR stimulation not only inhibit CD4^+^ T cell expansion, but suppress IFN-γ production by CD4^+^ T cells.

### CL097 is a potent inducer of CD4^+^ T cell death

**Fig 6:**
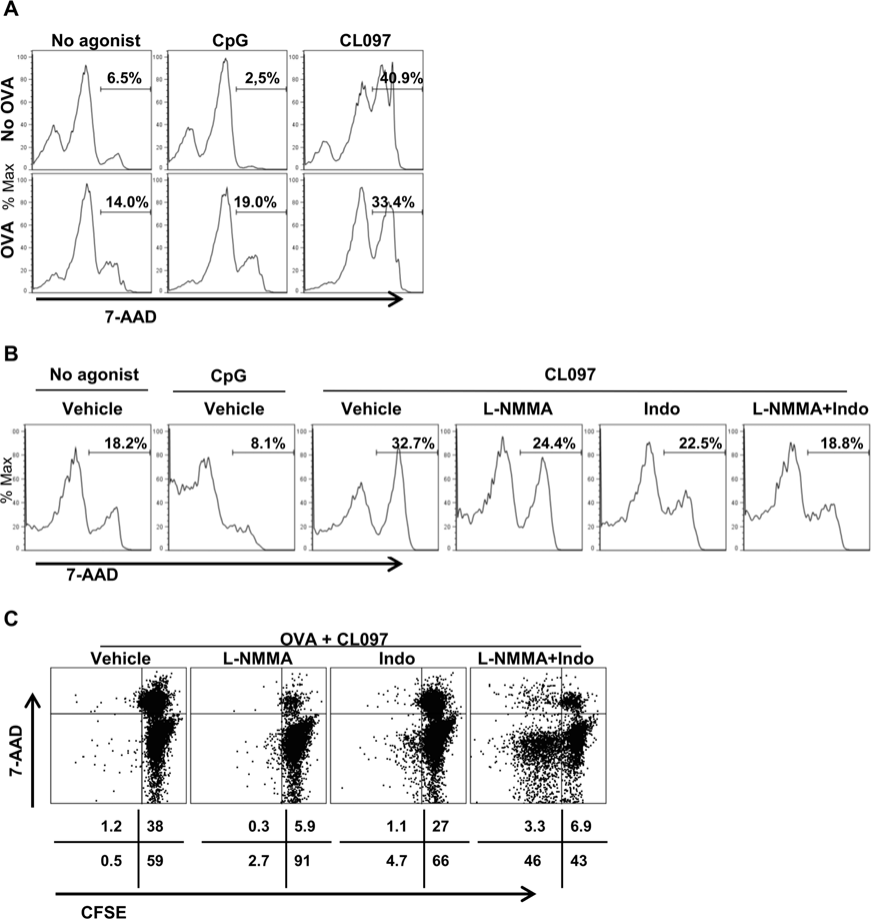
TLR7 stimulation causes elevation of CD4^+^ T cell death mediated by NO and PGE_2_. (A) Co-cultures of 5 x 10^5^ OT-II CD4^+^ T cells and 1 x 10^5^ DC were stimulated with 1 μg/ml CpG or 1 μg/ml CL097 in the presence or absence of 25 μg/ml OVA protein for three days. CL097, but not CpG, increased OT-II CD4^+^ T cell death regardless of antigenic stimulation. (B) 1 x 10^6^ splenocytes from wild-type mice were mixed with 1 x 10^5^ DC and then stimulated with CL097 in the presence or absence of L-NMMA or Indo for three days. CD4^+^ T cell death was reduced by L-NMMA and Indo. (C) Co-cultures of 5 x 10^5^ OT-II CD4^+^ T cells stained with CFSE and 1 x 10^5^ DC were stimulated with OVA protein plus CL097 in the presence or absence of L-NMMA or Indo for four days. Data are representative of two independent experiments.

Because both NO and PGE_2_ have been implicated in cell death pathways [42,43], we investigated if these modulators induced by TLR agonists have any effects on CD4^+^ T cell viability. OT-II CD4^+^ T cell/DC co-cultures were stimulated with CpG or CL097 in the presence or absence of OVA protein for three days, and the viability of CD4^+^ T cells were measured by 7-AAD staining. Interestingly, stimulation with CL097 without OVA potently increased, whereas CpG decreased 7-AAD-positive dead OT-II CD4^+^ T cells, compared to no agonist control (Fig 6A). To investigate if this CL097-induced cytotoxicity also occurs in non-transgenic wild-type CD4^+^ T cells, splenocytes of wild-type mice were cocultured with DC and stimulated with CpG or CL097 without OVA. Under this culture condition, stimulation with CL097 also reduced CD4^+^ T cell viability, relative to CpG and no agonist control (Fig 6B). In addition, CL097-induced CD4^+^ cell death was rescued equally by L-NMMA and Indo. The source of PGE_2_ in the absence of antigenic stimulation can be DC production of PGE_2_ (Fig 4A and 4B) and we also found that CD4^+^ T cell/DC cocultures stimulated with CL097 in the absence of OVA produced higher levels of IFN-γ than stimulation with CpG (S2 Fig), which is thought to elicit NO as well as PGE_2_. In the presence of OVA, addition of CL097 induced higher CD4^+^ T cell deaths than addition of CpG, which slightly increased the 7-AAD-positive cells (Fig 6A). Under culture conditions with OVA, both L-NMMA and Indo reduced OT-II CD4^+^ T cell death caused by CL097 with L-NMMA being more potent. These results suggest that CD4^+^ T cell death by PGE_2_ and NO may constitute mechanisms for the different adjuvanticity of CpG and CL097 (Fig 1).

### CL097 drives T cell expansion in the presence of L-NMMA and Indo in 2-week cultures

**Fig 7:**
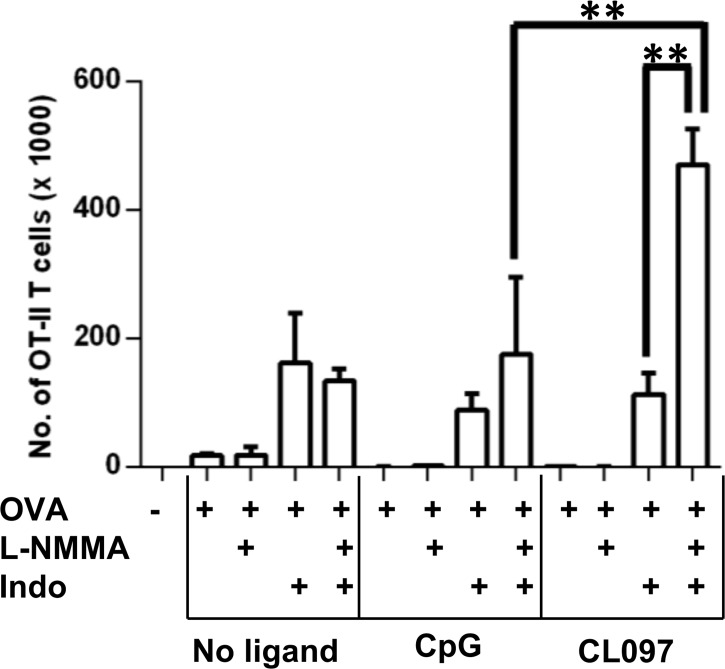
Simultaneous inhibition of COX-1/COX-2 and iNOS synergistically enhances OT-II CD4^+^ T cell proliferation stimulated with OVA protein plus CL097 in 2-week culture. 5 x 10^3^ OT-II CD4^+^ T cells were mixed with 1 x 10^5^ congenic DCs, and incubated with a combination of OVA protein, CpG, CL097, L-NMMA (40 μM), and Indo (10 μM) for 2 weeks to measure long-term CD4^+^ T cell expansion *in vitro*. At the end of the culture period, 4 x 10^5^ splenocytes from WT C57BL/6 mice (CD45.2) were spiked into the cultures to calculate the number of OT-II CD4^+^ T cells (CD45.1). Cells were stained with anti-CD45.1 and anti-CD4 antibody to measure the expansion of OT-II CD4^+^ T cells. 7-AAD was used to exclude dead cells in the analysis. Shown are mean numbers of OT-II CD4^+^ T cells of triplicate wells ± SD. Statistical differences were analyzed by one-way ANOVA. Data are expressed as mean ± SD of triplicate wells. Data shown are representative of three independent experiments. **p<0.001. iNOS: inducible nitric oxide synthase; COX: cyclooxygenase.

The CFSE dilution assay uses 5 x 10^5^ OT-II CD4^+^ T cells, which might not reflect the conditions of T cell activation *in vivo* where the frequency of antigen-specific T cell precursors is low. To model a more physiologic condition, we set up co-cultures with 5 x 10^3^ OT-II T cells. The OT-II/DC co-cultures were stimulated for 2 weeks with OVA in the presence or absence of the TLR agonists and iNOS/COX inhibitors. Following the incubation period, 4 x 10^5^ splenocytes from C57BL/6 mice (CD45.2^+^) were added to the culture to allow for the enumeration of CD45.1 OTII CD4^+^ T cells by the ratio of CD45.1^+^ to CD45.2^+^ CD4^+^ T cells. Results from the 2 week T cell proliferation system did not exactly reproduce the short-term T cell proliferation results measured by CFSE dilution. As shown in Fig 7, the proliferation of OT-II T cells was low in the absence of the inhibitors regardless of OVA and TLR ligand stimulation. During the 2-week incubation, Indo dominantly improved the expansion of OT-II T cells in all stimulations. Dual inhibition of NO and PGE_2_ synergistically enhanced CD4^+^ T cell expansion only when stimulated with OVA plus CL097, but not with OVA alone or with OVA plus CpG. This suggests that CL097 may exert adjuvant activity stronger than CpG to enhance CD4^+^ T cell expansion when both prostaglandins (PGs) and NO are inhibited.

### Neither PGE_2_ nor NO inhibition enhances the expansion of CD4^+^ T cells *in vivo*

**Fig 8:**
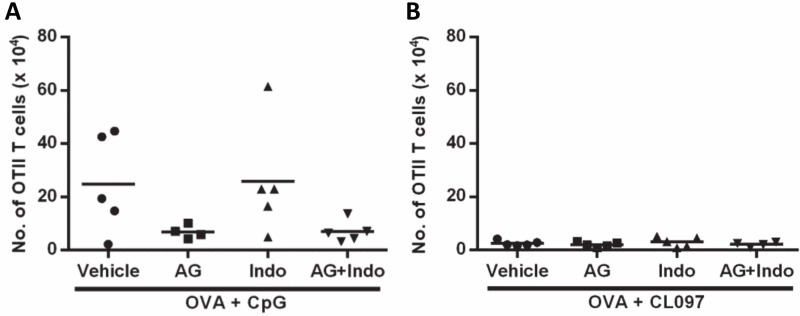
Expansion of OT-II CD4^+^ T cells are not enhanced by systemic treatment with Indo or AG. Mice were adoptively transferred with 1 x 10^6^ splenocytes obtained from OT-II mice and, 24 h later, immunized with 25 μg OVA protein with or without 20 μg CpG (A) or the same dose of CL097 (B) formulated in IFA. To inhibit NO, mice were given drinking water containing 2.5% AG and 1% glucose. To inhibit PGE_2_, mice were injected i.p. with Indo at a dose of 2.5 mg/Kg on the day of immunization and two more injections after 4 and 8 days. Mice were sacrificed 11 days post immunization, and the number of expanded OT-II CD4^+^ T cells were quantified by cell surface staining of CD4 and CD45.1 followed by flow cytometric analysis. The total numbers of OT-II CD4^+^ T cells in spleens were calculated based on the percentages of OT-II CD4^+^ T cells and known numbers of splenocytes. Statistical differences were analyzed by one-way ANOVA between treatment groups. *p<0.05. AG: aminoguanidine hemisulfate; Indo: indomethacin.

Our *in vitro* observations indicate that higher induction of PGE_2_ and NO by CL097 than CpG may account for the low adjuvanticity of CL097 compared to CpG (Fig 1). To investigate the *in vivo* relevance of our *in vitro* data, we treated mice with AG and Indo to inhibit NO and PGE_2_ production, respectively. AG was given in drinking water [28] and Indo was injected i.p. three times at four day intervals [27]. Unlike the *in vitro* observation that inhibiting both NO and PGE_2_ synthesis enhanced CD4^+^ T cell expansion, neither Indo nor AG treatment enhanced CD4^+^ T cell expansion after immunization with OVA plus CpG (Fig 8A) or CL097 (Fig 8B). This negative result with systemic inhibition may reflect the complexity of NO and PGE_2_ signaling pathways operating in a more complex *in vivo* environment.

## Discussion

Natural and synthetic adjuvants are being actively discovered and developed to support a growing trend preferring better defined subunit vaccines over live/whole vaccines [15]. However, there is currently no strong evidence to inform the rational selection of specific PRR agonists as adjuvants for a particular vaccine. We found that CpG, as an adjuvant, promoted stronger CD4^+^ T cell expansion than CL097 in mice. This difference in adjuvant potential of CpG and CL097 was delineated in the *in vitro* studies demonstrating that CpG was a weaker inducer of NO and PGE_2_ than CL097. This study provides evidence that adjuvant activity of PRR agonists is influenced by concurrent induction of regulatory factors. When comparing different PRR agonists, differential expression of regulatory products, such as NO and PGE_2_, should be considered and might be utilized as biomarkers of adjuvanticity.

One strategy to enhance vaccine efficacy is to increase the size of memory T cell populations. Because the size of memory T cell pools is proportional to the level of initial clonal expansion after antigenic stimulation [44], increasing the magnitude of the T cell expansion has been suggested to enhance memory generation [29]. Thus, we measured the expansion of antigen-specific CD4^+^ T cells to evaluate the adjuvanticity. We found that the adjuvant activity of the TLR9 ligand CpG was 10 times more potent than the TLR7 ligand CL097 in mice immunized with OVA. This result demonstrates that there exists a hierarchy among TLR agonists in promoting CD4^+^ T cell expansion that warrants further studies to define relative adjuvant activities of TLR agonists, which would enable informed decisions on which TLR agonists are selected for an adjuvant formulation.

To identify determinants accounting for the difference in adjuvanticity between CpG and CL097, we set up an *in vitro* co-culture of OT-II CD4^+^ T cells and DCs to model CD4^+^ T cell priming *in vivo*. TLR stimulation is required for DC maturation that drives antigen-specific T cell expansion *in vivo* [45]. Despite this requirement, BMDCs in our *in vitro* T cell priming assay were capable of driving antigen-specific T cell expansion without TLR stimulation, and the addition of CpG and CL097 paradoxically inhibited OVA-specific T cell proliferation as determined by CFSE dilution. This discrepancy can be explained in the nature of the DC and TCR-transgenic CD4^+^ T cells used for these co-cultures. DCs differentiated from bone marrow progenitors in media containing GM-CSF are highly efficient antigen-presenting cells with high basal expression of MHC II and costimulatory molecules [46]. BMDCs produced in this manner are capable of driving the expansion of even Treg cells, which are known to be anergic otherwise [47]. Nevertheless, the current *in vitro* system provided novel insight into the roles of suppressive signals on adjuvant activities.

We found that CL097 more strongly suppressed OVA-specific T cell expansion than CpG in the *in vitro* culture system, which could account for the lower adjuvanticity of CL097 that we observed *in vivo*. Factors that determine the differential suppression of T cell expansion by CpG and CL097 might predict adjuvanticity. Interestingly, this differential suppression of CD4^+^ T cell expansion directly correlated with IL-2 concentration in the cultures. Because IL-2 is a critical growth factor for T cell proliferation [48], we considered whether suppression of IL-2 was responsible for the suppression of the CD4^+^ T cell expansion in our culture system. Reconstitution and blocking experiments showed that this was not the case and, conversely, that IL-2 acted as a negative regulator of CD4^+^ T cell expansion in the cultures. It has been shown that IL-2 has both immunostimulatory and immunosuppressive roles depending on the context [49]. The immunosuppressive effect conferred by IL-2 is to potentiate Fas-induced activation-induced cell death [50,51]. This is a possible explanation for our *in vitro* results since we observed that adding exogenous IL-2 reduced the viability of CD4^+^ T cells, while neutralizing endogenous IL-2 enhanced viability as determined by 7-AAD staining. On the other hand, reconstitution or blocking of IL-2 did not restore CD4^+^ T cell expansion suppressed by CL097 stimulation, indicating that other regulators are involved. It is still to be determined whether IL-2 suppression by TLR agonists is an important issue in terms of their potential as vaccine adjuvants.

To identify immune components responsible for the suppression of CD4^+^ T cell expansion induced by the TLR agonists, we considered NO and PGE_2_ because they are recognized as negative regulators of T cell expansion [30–39]. As determined by CFSE dilution, the cell cycle arrest imposed by CpG and CL097 was partially lessened by either L-NMMA or Indo and the combination of the two inhibitors synergistically restored CD4^+^ T cell proliferation. This synergism of iNOS/COX double inhibition indicates that NO and PGE_2_ non-redundantly suppress T cell proliferation, which is consistent with the proposed mechanisms for these regulatory activities. Bingisser et al. [35] reported that suppression of T cell proliferation by NO is due to dephosphorylation of Janus kinase 3, and the signal transducer and activator of transcription 5, activation of guanylate cyclase, and resulting cyclic GMP production. On the other hand, PGE_2_ was shown to inhibit T cell activation by increasing cyclic AMP which inhibits cytosolic increases in [Ca^2+^] [52] and diacylglycerol and inositol phosphate production [53,54]. The observation that CL097 more potently induced NO and PGE_2_ than CpG is intriguing and might account for the poor *in vivo* adjuvanticity of CL097 compared to CpG.

The synergistic enhancement of IFN-γ production by inhibition of NO and PGE_2_ reflects the complex regulatory roles of the metabolites. It has been shown that IFN-γ stimulation induces both NO and PGE_2_ [40,41], and conversely, NO and PGE_2_ suppress IFN-γ production [55,56]. Based on these observations, a series of events involving IFN-γ and NO/PGE_2_ in inflammation can be postulated. As NO and PGE_2_ accumulate in inflamed tissues, IFN-γ levels likely decline due to the negative feedback loop. Reduced IFN-γ levels would in turn reduce NO production. Unlike NO, there exists a positive feedback mechanism between PGE_2_ [41] and COX-2, which may sustain PGE_2_ levels. Further studies are needed to define the dynamics of PGE_2_, NO, and IFN-γ levels in the context of vaccination and chronic infections.

Potent induction of CD4^+^ T cell death by CL097 has important implications in the use of TLR7 agonists as a vaccine adjuvant. This observation is not limited to murine cells because stimulation of human PBMCs with imiquimod induced higher CD4^+^ T cell turnover than other TLR agonists including LPS [57]. In the study, authors found that TLR7 stimulation preferentially killed CD4^+^ T cells over CD8^+^ T cells. We elucidate in this report that CL097-induced CD4^+^ T cell death was mediated by both NO and PGE_2_. These results warrant further investigation into whether CD4^+^ T cell death by TLR7 agonists through NO and PGE_2_ have implications in vaccine efficacy. Of particular interest, TLR7 stimulation induced CD4^+^ T cell death, whereas TLR9 stimulation is protective in the absence of antigen stimulation, This indicates that the adjuvanticity of a TLR agonist may be determined prior to antigen stimulation through modulation of CD4^+^ T cell turnover.

In the CD4^+^ T cell proliferation assay measured by CFSE dilution, we used 5 x 10^5^ OT-II CD4^+^ T cells per well and cultured them for 3–4 days. High numbers of monoclonal, transgenic CD4^+^ T cells in *in vitro* cultures may create too much competition for antigen stimulation and the cultures cannot be maintained > 6 days due to overpopulation. To generate a culture condition closer to the *in vivo* milieu, we set up a long-term OT-II T cell stimulation assay with a low starting number of cells (5 x 10^3^ cells per well) that ran for 2 weeks. Results from the long-term cultures were different from (and not predicted by) the data generated using higher initial numbers of CD4^+^ T cells in short-term culture. First, OT-II CD4^+^ T cells in long-term cultures that were stimulated with OVA stopped proliferating early regardless of the presence of the TLR agonists. This early termination of expansion was not due to depletion of nutrients or antigen, because media was replaced during the culture period and OT-II CD4^+^ T cells continuously proliferated in the presence of Indo. Unlike the short-term CFSE dilution experiments where L-NMMA alone increased proliferation of OT-II cells in the presence of OVA, iNOS inhibition had no impact on CD4^+^ T cell expansion in long-term cultures stimulated with OVA alone or OVA plus CpG or CL097. Furthermore, the synergistic elevation of CD4^+^ T cell expansion by L-NMMA plus Indo that was observed in the short-term culture system with OVA in combination with CpG or CL097 was only observed in the long-term cultures stimulated with OVA and CL097 together. The basis for these differences between the two culture conditions is presently unknown but may reside in the interaction of NO and PGE_2_ synthetic pathways, higher stability of PGE_2_ than NO, and positive feedback loop between PGE_2_ and COX-2 [41,58,59]. Whatever mechanisms are involved, these experiments revealed the potential for simultaneous inhibition of iNOS and COXs to enhance the adjuvant activity of TLR agonists.

Under *in vivo* conditions, there are multiple cell types contributing and responding to NO and PGE_2_ that may impact on adaptive immune responses after vaccination. There is constitutive, low level production of NO by neuronal, endothelial nitric oxide synthase, and prostanoids by COX-1 to conduct essential physiologic functions and homeostasis. Under inflammatory conditions, iNOS and COX-2 are induced in immune cells and generate a large amount of NO and PGs [60,61]. After vaccination, the first wave of NO/PGE_2_ production comes from leukocytes including neutrophils, monocytes, macrophages, and DCs, recruited to the injection sites. These cells transport antigens into draining lymph nodes for the initiation of the adaptive immune response [62]. Thus, a second wave of NO/PGE_2_ production takes place in the draining lymph nodes mediated by recruited and lymph node resident leukocytes. If a vaccine material is designed to be directly delivered to lymph nodes through lymph ducts, resident cells in lymph node would be the major source of the metabolites.

Despite the strong *in vitro* evidence, neither Indo nor AG treatment enhanced CD4^+^ T cell expansion in mice immunized with OVA plus CpG or OVA plus CL097. The failure to improve CD4^+^ T cell proliferation may be explained by diverse, often opposing effects that NO and PGE_2_ each exert in the immune system [61,63,64]. For example, PGE_2_ enhances influx of macrophages, neutrophils, and DC to injection sites, and DC activation and migration to draining lymph nodes, while it inhibits DC antigen presentation and T cell priming, and promotes regulatory CD4^+^ T cells and myeloid derived suppressor cells [60,63,64]. Likewise, NO has been shown to have pro-inflammatory/anti-inflammatory, pro-proliferative/anti-proliferative, and pro-apoptotic/anti-apoptotic properties in immune cells [43,65–69]. Thus, non-targeted, systemic inhibition of NO/PGE_2_ production may not be an optimal way to determine the role of mediators in adjuvant activities because the benefits of NO/PGE_2_ inhibition may be offset by the loss of the immunostimulatory effects of NO/PGE_2_.

The poor performance of CL097 as an adjuvant, as compared to CpG in our study is in contrast to the current interests in promoting TLR7 agonists as vaccine adjuvants. For humans, TLR7/8 agonists have an advantage over TLR9 agonists because TLR7/8 are expressed on multiple DC subsets, whereas TLR9 is expressed only in plasmacytoid DCs [70]. The adjuvant activity of CpG ODN has been well established in preclinical studies [15], and CpG is being tested in clinical studies as a component of IC31 and AS15 [71]. On the other hand, there is not enough evidence to determine the utility of TLR7/8 as a target for a vaccine adjuvant [15] despite that the TLR7 agonist imiquimod has been licensed as a topical formulation to treat genital warts, actinic keratosis, basal cell carcinoma, and lentigo maligna [72]. Considering the weak adjuvanticity of CL097 in our hands, it is conceivable that TLR7/8 agonists may be more suitable for therapeutic than prophylactic use. However, the utility of TLR7/8 agonists as an adjuvant should be evaluated using more optimized TLR7/8 agonists in diverse vaccine formulas and injection routes.

In summary, we show that the ultimate adjuvant potential of CpG and CL097 may be determined by their differential induction of immunoregulatory factors like NO and PGE_2._ The synergistic enhancement of T cell expansion by dual inhibition of iNOS and COX-1/COX-2 *in vitro* may also reflect endogenous mechanisms that blunt T cell immunity to infectious diseases with chronic inflammation, such as TB [73], and might provide potential targets for host-directed therapies. In the context of vaccination, the generation of NO and PGE_2_ may limit the efficacy of vaccines designed to boost cell-mediated immunity. Inhibiting these mediators in a way that enhances vaccine-induced immune priming might enhance protective efficacy against diseases like TB where traditional vaccination strategies have so far been disappointing.

## Supporting Information

S1 Fig CpG and CL097 equally enhance OT-II CD4^+^ T cell expansion to OVA stimulation up to four days post immunization. Splenocytes from OT-II mice stained with CFSE were adoptively transferred to WT mice at 1 x 10^6^ per mouse. The WT mice were then immunized s.c. with OVA formulated in IFA in the presence or absence of CpG or CL097. Four days later, inguinal lymph nodes were collected to examine the proliferative responses of OT-II CD4^+^ T cells. The two sets of ranges and numbers in each histogram represent cells that proliferated more than seven times (upper) and at least one time (lower).
(TIFF)

S2 Fig CL097 induces higher IFN-γ than CpG in the absence of antigenic stimulation. Purified CD4^+^ T cells of OT-II mice were co-cultured with DCs in the presence of CpG or CL097 for four days. IFN-γ levels were measured from culture supernatants by ELISA. The graph shows the mean ± SD of triplicate wells. Data are representative of four independent experiments**p<0.005.
(TIFF)
