# Supplementary figures and images for: Differential Adjuvant Activities of TLR7 and TLR9 Agonists Inversely Correlate with Nitric Oxide and PGE2 Production
Source: PLoS One. 2015 Apr 13;10(4):e0123165. doi: 10.1371/journal.pone.0123165 (PMC4395302; doi:10.1371/journal.pone.0123165)

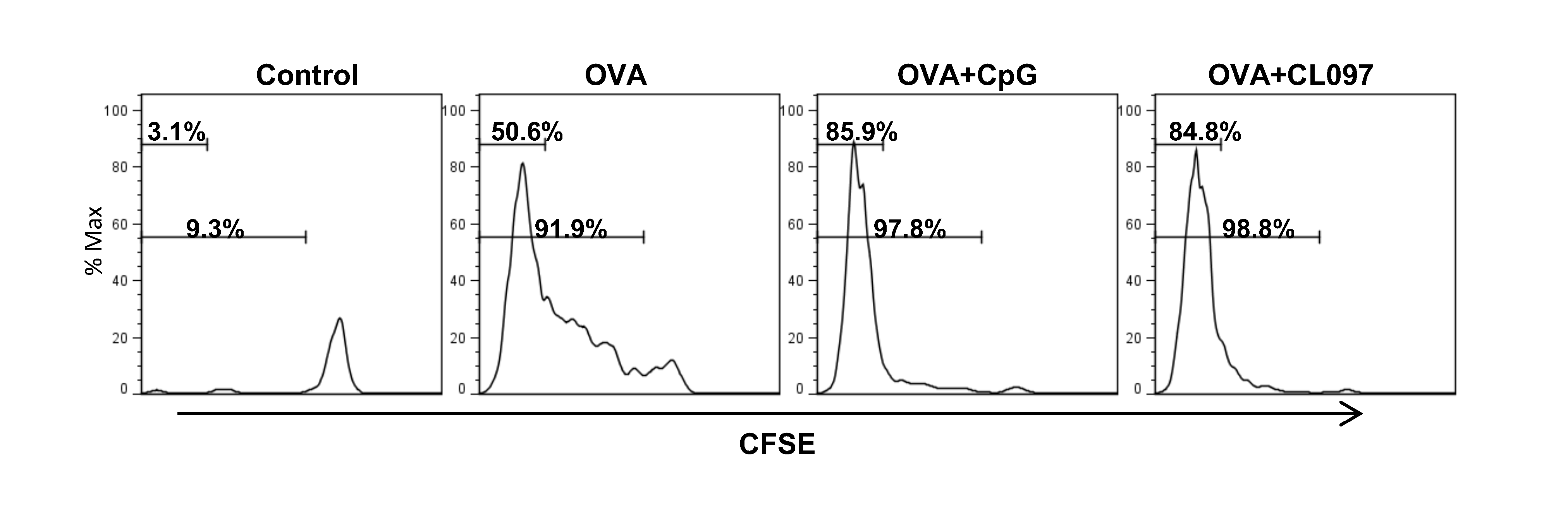

Supplement: S1 Fig — Splenocytes from OT-II mice stained with CFSE were adoptively transferred to WT mice at 1 x 10^6^ per mouse. The WT mice were then immunized s.c. with OVA formulated in IFA in the presence or absence of CpG or CL097. Four days later, inguinal lymph nodes were collected to examine the proliferative responses of OT-II CD4+ T cells. The two sets of ranges and numbers in each histogram represent cells that proliferated more than seven times (upper) and at least one time (lower). (TIFF) [file pone.0123165.s001.tiff]

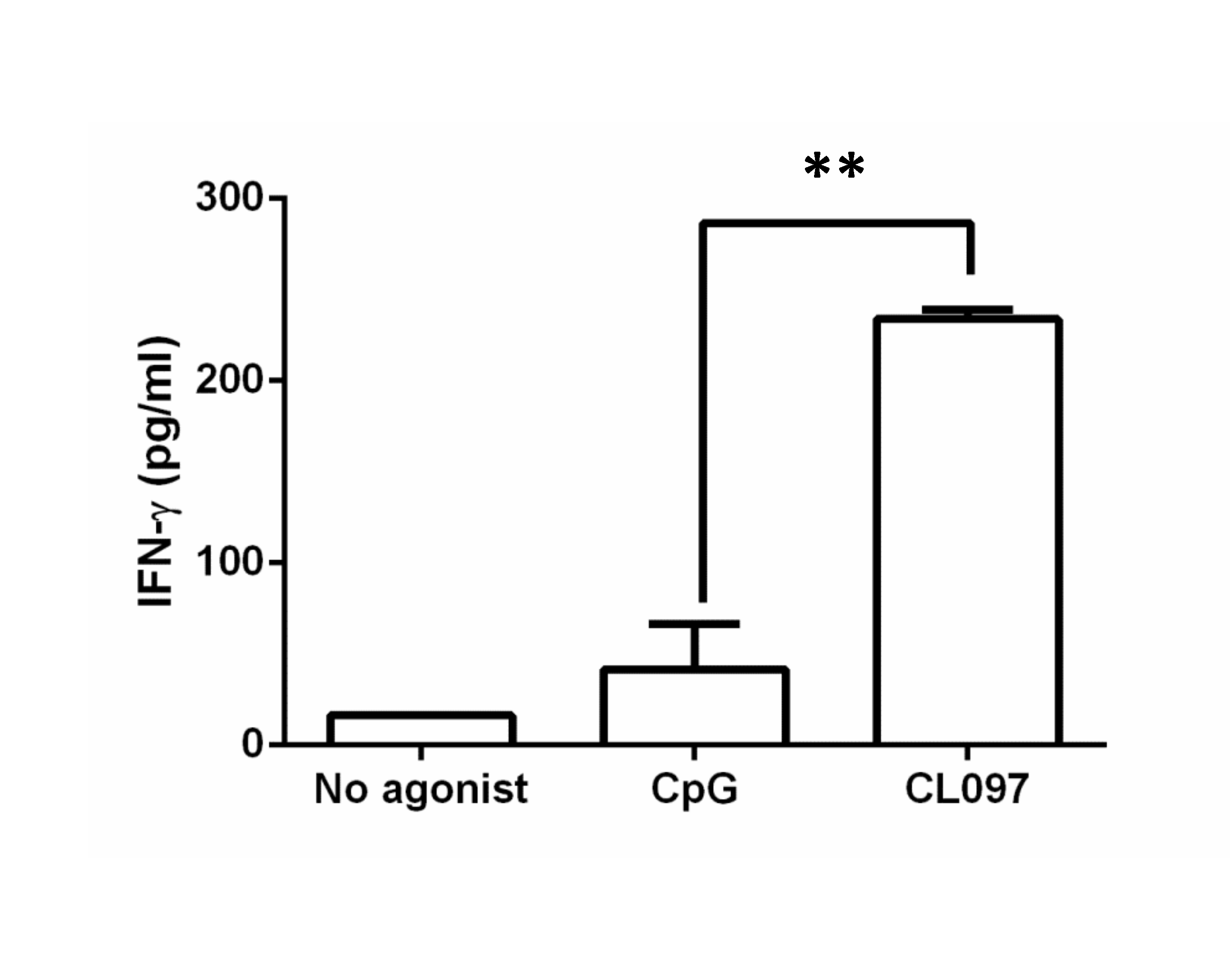

Supplement: S2 Fig — Purified CD4+ T cells of OT-II mice were co-cultured with DCs in the presence of CpG or CL097 for four days. IFN-γ levels were measured from culture supernatants by ELISA. The graph shows the mean ± SD of triplicate wells. Data are representative of four independent experiments**p<0.005. (TIFF) [file pone.0123165.s002.tiff]
